# Opportunities, approaches and challenges to the engagement of citizens in filling small water body data gaps

**DOI:** 10.1007/s10750-022-04973-y

**Published:** 2022-08-31

**Authors:** M. Kelly-Quinn, J. N. Biggs, S. Brooks, P. Fortuño, S. Hegarty, J. I. Jones, F. Regan

**Affiliations:** 1 School of Biology and Environmental Science & UCD Earth Institute, Dublin, Ireland; 2 Freshwater Habitats Trust, Oxford, UK; 3 Department Life Sciences, Natural History Museum, London, UK; 4 FEHM (Freshwater Ecology, Hydrology and Management), Department of Evolutionary Biology, Ecology and Environmental Sciences, & Institut de Recerca de la Biodiversitat (IRBio), University of Barcelona, Barcelona, Spain; 5 DCU Water Institute, Dublin City University, Dublin, Ireland; 6 Queen Mary University of London, London, UK; 7 School of Chemical Sciences, Dublin City University, Dublin, Dublin, Ireland

**Keywords:** Small water bodies, Citizen science, Small streams, Ponds, Catchment types, Framework, River condition assessment

## Abstract

Monitoring the condition (water quality, biodiversity, hydromorphology) of small water bodies presents a challenge for the relevant authorities in terms of time and resources (labour and financial) due to the extensive length of the stream network or the sheer number of small standing water bodies. Citizen science can help address information gaps, but the effort required should not be underestimated if such projects are to generate reliable and sustained data collection. The overall aim of this paper is to propose a framework for operationalisation of citizen science targeting collection of data from small water bodies. We first consider the data gaps and the elements (water chemistry, ecology, hydromorphology) to be addressed, in order to define where citizen science could best make an impact. We review examples of tools and methods that are appropriate for small water bodies, based on experience from a selection of freshwater citizen science projects, and the support that is needed for effective and sustained small water body projects across Europe.

## Introduction

The predominance of small water bodies in the landscape, their significance in terms of biodiversity and the influence of 1st and 2nd order streams on catchment water quality are well recognised (e.g. Biggs et al., 2017; Riley et al., 2018). However, most are not covered by the Water Framework Directive (2000/60/EC; WFD) monitoring which requires river water bodies to have a catchment area greater than 10 km^2^ and lakes to have an area of > 50 ha. Consequently, small waters largely remain the least monitored freshwater resources with significant gaps in terms of spatial and temporal coverage. For example, in Ireland less than 10% of the river sites in the national water quality monitoring programme are on small streams. At the same time small streams, as well as other small water bodies such as ponds, are highly susceptible to anthropogenic pressures due to high connectivity with adjacent land and low dilution capacity.

Monitoring the condition (water quality, biodiversity, hydromorphology) of small water bodies presents a challenge for the relevant authorities in terms of time and resources (labour and financial) due to the extensive length of the stream network (e.g. 63,731 km in Ireland—75% of the length of the river network (Kelly-Quinn & Reynolds, 2020), c. 3 million km in EU member states (Kristensen & Globevnik, 2014) or the sheer number of small standing water bodies (e.g. an estimated 478,000 ponds in Great Britain; Williams et al., 2010). Hence, citizen science is becoming increasingly important in environmental research and monitoring (e.g. Silvertown, 2009), including river environments (e.g. DiFiore & Fitch, 2016; Shuker et al., 2017), as it has the potential to fill some of the data gaps relating to small water bodies.

The term citizen science and what it aims to achieve have been variably defined. Citizen science is amongst a plethora of terms that relate to various aspects of public participation in areas of scientific investigation, as diverse as species recording, air monitoring and astronomy. Two people defined the term independently in the mid-1990s. Social scientist Alan Irwin used it in the UK to emphasise that science should be responsible to citizens' needs and that citizens themselves could produce reliable scientific knowledge (Irwin, 1995). Rick Bonney, in the USA, defined it as a ‘research technique on which non-scientists voluntarily contribute scientific data to a project’ (Bonney et al., 2009). Other definitions refer to the involvement of citizens in the co-design of projects and data analysis (Haklay et al., 2021). Despite the variable definitions, the core elements of citizen science are the inclusion of non-professionals in data collection and the use of these data to address genuine scientific objectives. Apart from data collection, citizen science projects have a number of other advantages and added value particularly through establishing societal awareness, improving people’s connection with the natural environment and its protection (Science Communication Unit, 2013), as well as the potential to contribute to policy making (Schade et al., 2021). As noted by Gurnell et al. (2019), the contribution of citizen science monitoring of environmental conditions has grown rapidly in recent years. Projects relating to rivers were noted as too numerous to quantify, occurring on all continents, but are most numerous and longest established in North America, Europe and Australia.

The overall aim of this paper is to propose a framework for operationalisation of citizen science targeting collection of data from small water bodies. We first consider the data gaps and the elements (water chemistry, ecology, hydromorphology) to be addressed, in order to define where citizen science could best make an impact. We review examples of tools and methods that are appropriate for small water bodies, based on experience from a selection of freshwater citizen science projects (see Table 1) and the support that is needed for effective and sustained small water body projects across Europe. The definition of effectiveness is generally determined by the specific goals of the various projects, but in terms of the identified needs relating to small water bodies, effective citizen engagement should generate good quality data that address the identified gaps, and those data should be accessible for analysis and use by researchers and resource managers. The support and resources relating to these core elements, together with challenges and solutions needed to sustain engagement, will be highlighted by the citizen science initiatives described in this paper to help inform the proposed framework.

**Table 1:** Selected examples of citizen science initiatives at country, continent and global levels

No	Name of Initiative	Country	Water body targeted	Type of data	Training and resources	Data input (paper or online form)	Data Repository	Website	Suitability for small waters
1	Clean Water for Wildlife	UK	All (ponds, streams, rivers, lakes, ditches, fens, springs, flushes)	Nitrate, phosphate	Workshops, tutorials, videos, written materials	Online	Freshwater Habitats Trust website	https://freshwaterhabitats.org.uk/projects/clean-water/	Yes
2	Anglers’ Riverfly Monitoring Initiative (ARMI)	UK	Rivers	Macroinvertebrates	Workshop and written materials	Online to national database	National database held at the Freshwater Biological Association	https://www.riverflies.org/recording-schemes	Yes
3	RiuNet	Spain	Small streams	Hydrology, riparian forest quality aquatic habitat heterogeneity Macroinvertebrates Ecosystem services	Tutorial in app, Training provided	Mobile app	FEHMLab	http://www.riunet.net	Methodology has been used on small streams
4	Projecte Rius	Spain	Small streams	Hydrology, riparian forest quality aquatic habitat heterogeneity Macroinvertebrate Temperature, nitrates, oxygen, pH, turbidity	Training provided, Instruction leaflet	Online form on website and mobile app	Associació Hàbitats	http://www.projecterius.cat	Methodology has been used on small streams
5	FreshWater Watch (incl. WaterBlitz)	Europe	All	Nitrate, phosphate	Tutorial in app, information leaflet provided	Mobile app	Freshwater Watch	http://www.freshwaterwatch.org	Methodology has been used on small streams
6	1000 punts d’aigua	Spain	Springs, ponds	Typology, hydromorphology, Flora, fauna, impacts Conservation status	Handbook, Instruction leaflet	Online form on website and mobile app	Paisatges Vius	https://1000punts.cat/ca/projecte-que-es-un-punt-d-aigua)	Developed for small water bodies from Catalonia region
7	MoRPh	UK and Ireland	Rivers and streams	Geomorphic features/indicators, physical habitats, riparian and in-channel vegetation structure, human pressures and interventions	Training courses, supporting written materials—online form, mobile app Also includes training for MoRPH trainers	Fully accessible On-line Information System	cartographer.io	https://modularriversurvey.org/	Yes, developed for wet or dry watercourses < 20 m wide
8	PondNet (Freshwater Habitats Trust)	UK	Ponds	Biological surveys of individual species, including eDNA	Tutorial, videos, written materials	Online	Freshwater Habitats Trust website	https://freshwaterhabitats.org.uk/projects/pondnet/	Standard survey methods developed for a range of species, and eDNA surveys
9	CSSI—Citizen Science Stream Index	Ireland	Small streams	Macroinvertebrates	Instruction leaflet Training workshops	In development	National Biodiversity Data Centre	https://lawaters.ie/citizen-science/	Developed for small streams
10	SSIS—Small stream Impact Score	Ireland	Small streams	Macroinvertebrates	Handbook Training workshops	In development	National Biodiversity Data Centre	https://lawaters.ie/citizen-science/	Developed for small streams
11	Dragonfly Ireland	Ireland and Northern Ireland	All freshwaters	Adult dragonflies	Workshops and identification guides	Online form	National Biodiversity Data Centre	https://biodiversityireland.ie/surveys/dragonfly-ireland/	Yes
12	Backdrop	Ireland	Urban river	Nitrates, phosphates, turbidity	Freshwater Watch	Online form on mobile app	Earthwatch (Freshwater Watch programme)	https://dcuwater.ie/backdrop/	Methodology has been used on small streams
13	Centennial	Ireland	Rural and urban rivers	Nitrates, phosphates, turbidity	DCU and Freshwater watch	Online form on mobile app	Earthwatch (Freshwater Watch programme)	In development	Methodology has been used on small streams
14	FreshWater Watch	Europe	All	Nitrate, phosphate	Tutorial in app, information leaflet provided	Mobile app	Freshwater Watch	www.freshwaterwatch.org	Methodology has been used on small streams
15	DRYRivERS	Europe	Intermittent rivers and streams	Hydrological: flow, pools or dry conditions	Tutorial and video	Online	University of Pécs	https://www.dryver.eu/citizen-science/introduction	Yes, many intermittent rivers are small water bodies
16	Plastic Origins	Europe	Rivers	Plastics	Online training	Mobile app	Plastic Origins website	https://www.plasticorigins.eu/	Yes
17	Secchi Dip-In	North America	Lakes, rivers & estuaries	Secchi readings	Online video	Online form	North American Lake Management Society	https://www.nalms.org/secchidipin/	Yes
18	CrowdWater	Global	Intermittent rivers and streams	Hydrological data	app tutorial, leaflets, gamification	Online form on mobile app	University of Zurich	https://crowdwater.ch	Yes, also has a focus on temporary streams
19	EyeOnWater	Global	Freshwater and marine surface waters	Surface water colour	Some on site	Mobile app	-	https://www.eyeonwater.org/home	Yes for standing water bodies

## A role for citizen science in monitoring small water bodies—data collection gaps?

The gaps in the data from the small stream network relate to physical and chemical, ecological and hydromorphological conditions. In some cases information is needed on the location of water bodies (e.g. 1000 punts d’aigua—Table 1) or condition of specific type of waters, e.g. intermittent streams (DRYRivERS project—Table 1) or specific events such as floods and droughts (e.g. CrowdWater—Table 1). There is also the potential to support biodiversity monitoring (Schmeller et al., 2009; Chandler et al., 2017) and plastic-pollution research (Cook et al., 2021; e.g. PlasticsOrigin project https://www.plasticorigins.eu/). Key physical and chemical measurements include nutrients (NO_3_ and PO_4_) in terms of eutrophication risk and deposited fine sediment, which is widely considered to be a master stressor (Blöcher et al., 2020). Related to the latter measurement is hydromorphological degradation, i.e. human-induced changes in the flow regime, channel modifications and geomorphic (physical habitat) complexity, which is becoming increasingly recognised as a stressor affecting ecological health and ecosystem functioning, with the potential to interact with other stressors (Elosegi et al., 2019). Biological indicators such as macroinvertebrates are especially important as measures of prevailing conditions. Citizen science schemes can deliver macroinvertebrate-based metrics describing valid water quality or pollution risk information that can be used by national or regional agencies. The potential contribution of citizen science to BACI (Before-After-Control-Impact) oriented monitoring of ecological restoration in streams and assessing effectiveness of mitigation measures are also recognised (Edwards et al., 2018) as well as detection of invasive species (Clusa et al., 2018).

There is clearly a need to gather more information on the biodiversity of small streams and other small water bodies, however, this is constrained by the level of taxonomic skill, the extent of training, time available and experience needed for the application of standard biological assessment methods that can be undertaken by citizens. This will require identification of key indicators by researchers before these gaps can be addressed more widely.

Data collection gaps for ponds are similar to those for the small stream network. Information needs include (a) assessment of the number of small ponds, (b) assessment of the overall condition of ponds and small lakes, (c) identification of water bodies that are of importance for biodiversity and (d) assessment of trends in the condition of water bodies and the status of specific protected species. Current approaches to these data gaps were reviewed specifically for the UK by Biggs et al. (2022) but are of wider relevance.

## Methods appropriate for citizen science monitoring in SWBs incl. lessons learned

A central challenge is identifying methods which can be easily and safely applied by citizen scientists yet produce data that are of sufficient quality to support practical monitoring of water quality, species and habitat assessment and inform decision making and policy (Hegarty et al., 2020). Here we consider options available for the collection of physical and chemical, ecological and hydromorphological data. Examples of projects mentioned and described later are included in Table 1.

### Physical and chemical monitoring

There are a limited number of physical and chemical measurements that can be used by citizens to generate data. These generally only give an indication of whether values are elevated or low with respect to what is normal, and therefore their limitations need to be recognised. Despite this, valuable indications of various aspects of water quality can be derived from citizen science data. For example, one of the longest running citizen science initiatives, Secchi Dip-In, has provided publishable data on long-term trends in water quality (Lottig et al., 2014). The EyeOnWater project (Table 1) uses surface water photos, and where available Secchi disk data, uploaded by citizens for calibration of satellite imagery.

Kits for nutrient analysis are amongst the most widely used to provide data on hydrochemical water quality. The nutrient kits consist of transparent plastic tubes, in which participants mix unfiltered water samples from sampling cups with pre-measured reagents. A colour-change in the tube varies with increasing concentration of nutrients (McGoff et al., 2017; Scott & Frost, 2017) and is compared visually to a six-point colour chart. The range of concentrations covered by the two closest colour matches is recorded as the test result (McGoff et al., 2017). Whilst the results of these kits are less precise than those measured using laboratory methods, they allow citizen scientists to obtain results quickly *in situ*, as well as being simple and safe for volunteers to use unsupervised. Citizens also note land use at the locations where they sample, as well as information on algae, litter and other visual details of the sample site (Thornhill et al., 2017). The Pack-Test kits have been chosen for use in a variety of situations both professionally and involving citizen scientists following a period of extensive testing (Thornhill et al., 2016). A technical guide to the use of the test kits has been produced by The Freshwater Habitats Trust (FHT) (Biggs et al., 2016). The two lowest categories (< 0.02 and 0.02–0.05 ppm for phosphate; < 0.2 and 0.2–0.5 ppm for nitrate) are generally equivalent to clean, or minimally impaired conditions and categories above these represent polluted conditions. However, they caution against this interpretation in that naturally oligotrophic waters may be impacted by concentrations of nutrients below these values. Overall, these kits are good at landscape level for spotting pollution hotspots but are not sensitive to small ecologically relevant temporal changes. They are most valuable where they provide data which are not already gathered by other methods, and where the resolution of the data provided is sufficient for meaningful decision making.

**Fig. 1:**
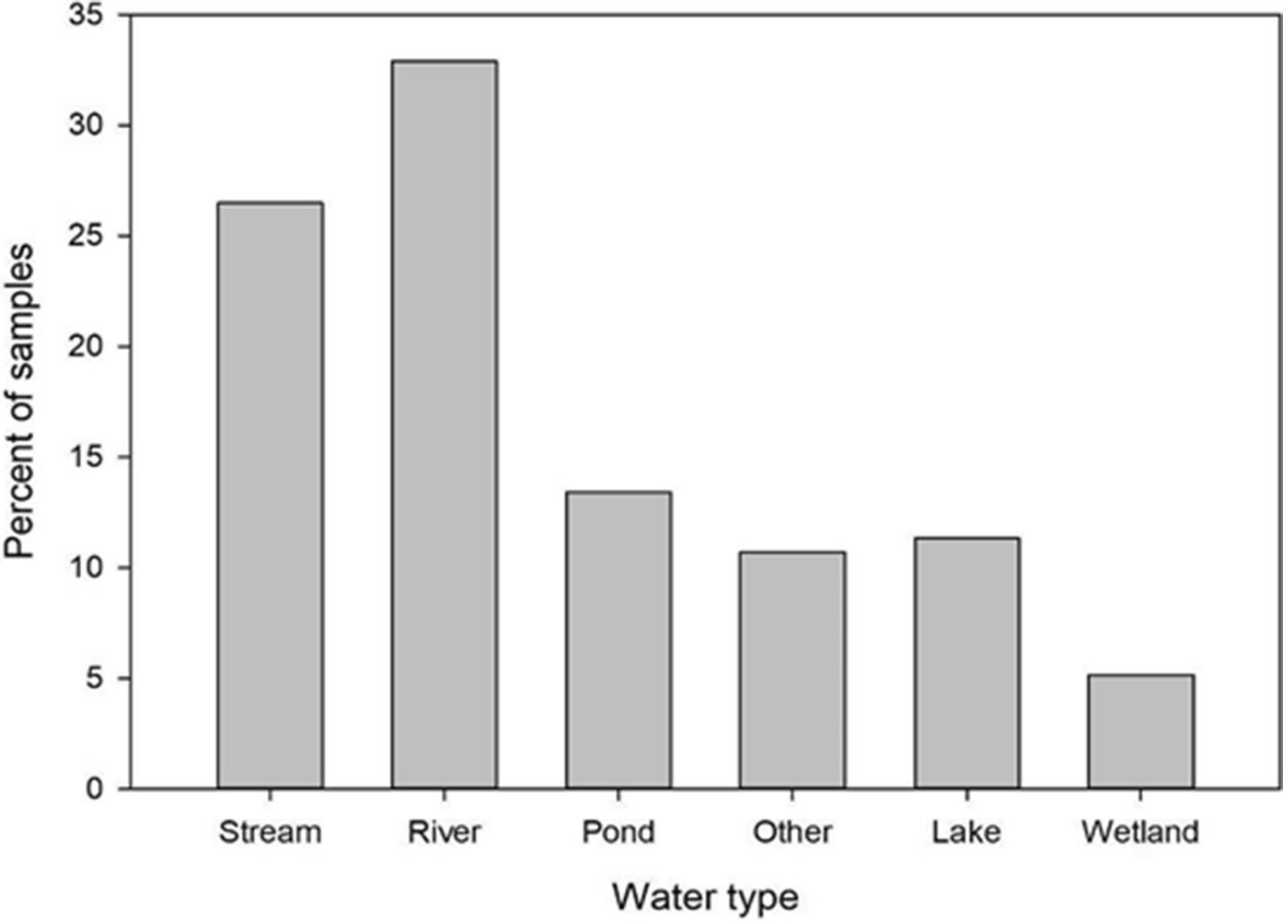
Percentage of samples taken from different water types by Freshwater Watch citizen scientists, 2013–2019. Data courtesy of Earthwatch from data gathered by FWW citizen scientists, mapped by authors. Based on “Earthwatch FreshWater Watch data” accessed 20/02/2020 by the authors, under CC BY. It is licensed under CC BY by Earthwatch Institute

At the global level, Earthwatch’s FreshWater Watch (FWW) has gathered over 30,000 data points since its foundation in 2012, each using the same methodology and platform, leading to data that can be comparatively analysed (Thornhill et al., 2016; Quinlivan et al., 2020; Hegarty et al., 2021). Within FWW, over 80 local projects in more than 20 different countries have been established to date (e.g. Dublin Water Blitz detailed later). Volunteers are generally attracted to the scheme through sampling their local small water bodies. Since its beginning in 2012, 27% of all FWW samples have been taken from streams, with a further 13% from ponds (Fig. 1).

In addition to taking measurements of nutrients, many FWW projects collect turbidity information using calibrated Secchi tubes distributed to the citizen scientists by the project team (cf. Miguel-Chinchilla et al., 2019). These tubes measure turbidity using the nephelometric turbidity scale, between 12 and 240 NTU. Laboratory analysis has shown that turbidity data gathered by citizen scientists using these FWW Secchi tubes are related to the amount of total suspended solids in the watercourse. However, in common with other citizen science methods, lower levels of turbidity are not measurable using this method, with over 50% of samples in one study being below 12 NTU (Scott & Frost, 2017). When turbidity can be measured, the data collected can inform researchers about the effect of land use change, such as urbanisation, on sediment loads in freshwater bodies. A paper by Lottig et al. (2014) reported the value of Secchi disc data collected by citizen science for over 3,000 lakes.

The use of citizen scientists to provide more detailed hydrochemical data is generally constrained by the availability of equipment and the training required to enable citizen scientists to maintain the equipment and interpret the data. However, at small spatial scales the two-way interaction between citizen and professional scientists can lead to better engagement and understanding of water quality issues, as exemplified by the Chesswatch project (http://www.riverchessassociation.co.uk/news/74/57/ChessWatch-A-River-Observatory.html) investigating a small chalk stream in suburban London, UK. Here, with support of citizen scientists, sensors record selected water quality indicators at thirty-minute intervals (including water level, dissolved oxygen, turbidity, chlorophyll-*a*) and is linked to other citizen science initiatives (see Anglers’ Riverfly Monitoring Initiative below). The aim of the project is to develop an informed consensus on the management of this small river by linking citizen scientists with regulators and water companies through a common understanding of the issues and pressures.

### Biological data

Many of the long-standing citizen science programmes have involved the collection of biological data, but these are often confined to a few countries and focussed on macroinvertebrate water quality indicators. For example, in the USA there are approximately 1700 volunteer water quality monitoring programmes (http://volunteermonitoring.org/) and according to Peeters et al. (2022), circa 50% of these involve macroinvertebrate indicators. Other well-established programmes noted by Peeters et al. (2022) include the British Anglers’ Riverfly Monitoring Initiative (described in more detail later) and the New-Zealand Wai Care programme (https://localgovernmentmag.co.nz/wai-care-programme/). Whilst monitoring water quality can be based at relatively high-level taxonomic identification (e.g. order or family), biodiversity studies involve identification to levels that require much longer training periods. However, it can be useful for selected invertebrate groups such as Odonata. For example, Dragonfly Ireland 2019–2024, is an all-Ireland citizen science survey of dragonflies and damselflies, and their habitats, coordinated by the National Biodiversity Data Centre in the Republic of Ireland, and by the Centre for Environmental Data and Recording in Northern Ireland. Interestingly, this project offers volunteers three levels of participation; *Spotters* submit casual records; *Recorders* conduct timed surveys and *Monitors* conduct repeated surveys (https://biodiversityireland.ie/surveys/dragonfly-ireland/). Training, including online modules, and identification swatches are provided, and an online record entry system is available. The data collected by the National Biodiversity Data Centre are available as Open Access data (CC-BY 4.0) via the Biodiversity Maps system. In Ireland, a recent citizen science initiative has provided two macroinvertebrate-based schemes (Table 1), the Citizen Science Stream Index (CSSI) based on 6 indicator taxa (Heptageniidae, any plecopteran, *Rhyacophila*, any snail, leech and *Asellus*) and the Small Stream Impact Score (SSIS) based on 5 groups of indicators (Ephemeroptera—7 key taxa, Plecoptera—7 key taxa, Trichoptera—8 key taxa, Gastropoda/Oligochaeta/Diptera—10 key taxa and *Asellus*). All participants start with the CSSI and those wishing to learn a wider range of taxa or to become trainers are instructed in the SSIS.

Surveying wetland plants is more challenging for the non-specialists, mainly because identification to species level, which is most useful, can be difficult and requires substantial training and experience. However, there is an enormous wealth of volunteer-generated data that have been produced by the Botanical Society of Britain and Ireland (https://bsbi.org/maps—e.g. Preston et al., 2002) and the British Bryophyte Society (https://www.britishbryologicalsociety.org.uk/learning/species-finder/—e.g. Blockheel et al., 2014) for distribution mapping indicating what is possible through coordination of enthusiastic and highly skilled citizen scientists. Such datasets provide vital information on the assessment of large-scale, long-term trends. Experienced surveyors can rapidly collect large wetland plant-based datasets, which are extremely cost-effective and have been used to demonstrate important landscape management results (e.g. Williams et al., 2004, 2020). In terms of amphibians, there is a substantial cadre of surveyors able to recognise the organisms and the main challenge is organising volunteers into effective surveys. This is because surveying amphibians often requires multiple site visits and may require time-intensive sampling methods like bottle trapping, which also bring significant animal welfare requirements. In the UK, for example, substantial effort has been put into organisation of citizen-led surveys of amphibians, initially in the National Amphibian and Reptile Recording Scheme (Wilkinson & Arnell, 2013) now relaunched as the National Amphibian and Reptile Monitoring Programme (see https://monitoring.arc-trust.org/). Despite the popularity of amphibians, it is often difficult to get people to visit enough sites, in the right places, without substantial organisational support. This is partly why the great crested newt eDNA project, described below, has been successful as it has enabled large numbers of sites to be surveyed comparatively quickly, resulting in a larger dataset than was possible with ‘traditional’ methods. Good data can be collected on water birds by citizen scientists and there are major survey programmes (e.g. Crowe et al., 2010; BTO, 2017; Harris et al., 2021;) which incorporate some small waters, although the emphasis is mainly on larger sites. Citizen based surveys are successful because of substantial long-term backing by NGOs and government agencies, as well as other ‘pro’ factors, such as the large pool of technically able volunteer ornithologists. Monitoring aquatic mammals is not particularly suited to citizen science because of the methods and skills that are needed.

### Hydromorphology

Hydromorphology describes flow regimes, the condition of morphological elements (e.g. substrate composition, channel morphology, bank condition), as well as channel form and connectivity (Elosegi et al., 2010). There is growing awareness that degraded hydromorphology impacts river ecosystem functioning and ecological health, and contributes to biodiversity losses (e.g. Elosegi & Sabater, 2013). Hydromorphological pressures are reported to impact 40% of European water river bodies (Kristensen & Walley, 2018), a figure that does not include small streams. These small water bodies have been extensively modified through channelisation, dredging, weed cutting, water abstraction and removal of riparian vegetation (e.g. Rasmussen et al., 2013). Many hydromorphological assessment methods have been developed for use by professionals (e.g. Belletti et al., 2015) but less attention has been given to a potential role for citizen science. Exceptions include CrowdWater, a citizen science project initiated by the University of Zurich, involving the collection of water level data with both physical and virtual staff gauges using a mobile app. Repeated photographs at ungauged sites help establish flow information, and citizens can use the advanced settings to estimate discharge. The project is also collecting data from temporary streams.

The modular river survey, MoRPH Rivers (Shuker et al., 2017), enables citizens to assess the condition of physical habitat in rivers (Table 1) where reaches or a “module” of river, approximately two channel widths in length, is surveyed. It captures data on channel morphology (including bankfull width, bank height and water depth), the presence of channel bars, channel composition and vegetation (bed and bank materials, invasive species present, littoral vegetation). According to Gurnell et al. (2019), 10 contiguous MoRPh modules cover the range of habitats available to more mobile species such as fish. Three levels of training are provided: introducing, reinforcing and MoRPH trainer training. Field survey data are stored in the Modular River Survey database and are freely available online. The web-based system generates values for 14 habitat indicators (see Gurnell et al., 2019). This tool is applicable to small streams including dry stream channels.

## Experience gained from selected case studies

In this section we provide insight into a number of citizen science projects that can inform a strategy for successful volunteer engagement. A selection of freshwater projects are summarised in Table 1. The key information compiled relates to the relevance of the projects to small water bodies, variables measured, training and data acquisition. Three well-established projects which have included small water bodies are described in more detail. One shorter-term WaterBlitz project is included because of its participants’ focus on small streams. A short background to the selected projects and their methodology are provided by way of introduction. The main focus is on the elements that have contributed to their success which can inform a framework for operationalising citizen science projects in small water bodies. Key elements highlighted by these projects and others in Table 1 are indicated by a project number in the framework described later.

### UK Freshwater Habitats Trust projects

#### Background and methodology

In the UK, The FHT has widely applied two approaches which, following detailed testing, have shown promise to provide data which allow a whole landscape approach to be adopted, are valuable for practical decision making, and provide data which are not otherwise available. These deploy ‘rapid’ nutrient pollution testing (PACKTEST kits) and the use of eDNA to detect a protected species, the great crested newt (described in a later section). Several large-scale projects evaluating nutrient pollution as an indicator of overall water quality in contrasting landscapes, known as ‘Clean Water for Wildlife’ have been run. These projects have provided (a) the first data in all of the areas studied which describe nutrient pollution in water bodies representative of the whole water environment, including small water bodies such as ponds, streams, rivers, lakes, ditches, fens, bogs, springs, flushes, (b) the first comparative water quality data on large and small water bodies and (c) a simple way of demonstrating the particular role of smaller waters as ‘refuges’ for ‘clean’ water in the landscape. In this case, ‘clean’ water has a precise definition, being as far as possible equivalent to High status in the WFD, allowing for the fact that nutrient testing kits cannot produce results with the same level of precision and sensitivity as laboratory water quality analyses. The methodology is summarised in Biggs et al. (2016). As far as possible surveys are undertaken in the spring or early summer with 70% of sites surveyed in the period March to June.

#### Training

Prior to participating in ‘Clean Water for Wildlife’ projects, volunteers are trained in the survey methods in workshops lasting half to one day, and the role of the survey in understanding impacts of nutrient pollution are explained. Training is conducted by members of FHT with standard explanatory resources, including written documents and video guides. Training courses are also provided online where circumstances require. During the workshops methods are demonstrated and participants test a range of nearby water bodies. The online recording system is demonstrated to participants.

Volunteers normally interact with a specific project coordinator who organises site permissions. As all surveys in the UK require the permission of the landowner, a substantial part of the work involves FHT staff planning survey locations (typically a stratified random sample of 1 km squares) and obtaining permission from landowners to visit sites.

#### Data capture

‘Clean Water for Wildlife’ survey results are captured in online forms embedded in the FHT website. A simple screening step is incorporated to ensure that results are correctly recorded. Recording of data has been designed for standard PC platforms. This is often as convenient as field observations because the optimum approach to the use of PACKTEST kits is to collect water samples in the field and then analyse samples in batches at the volunteer’s home or workplace where standard temperature (PACKTEST kits should be used at room temperature) and lighting conditions can be most easily provided.

#### Extent of results

**Fig. 2:**
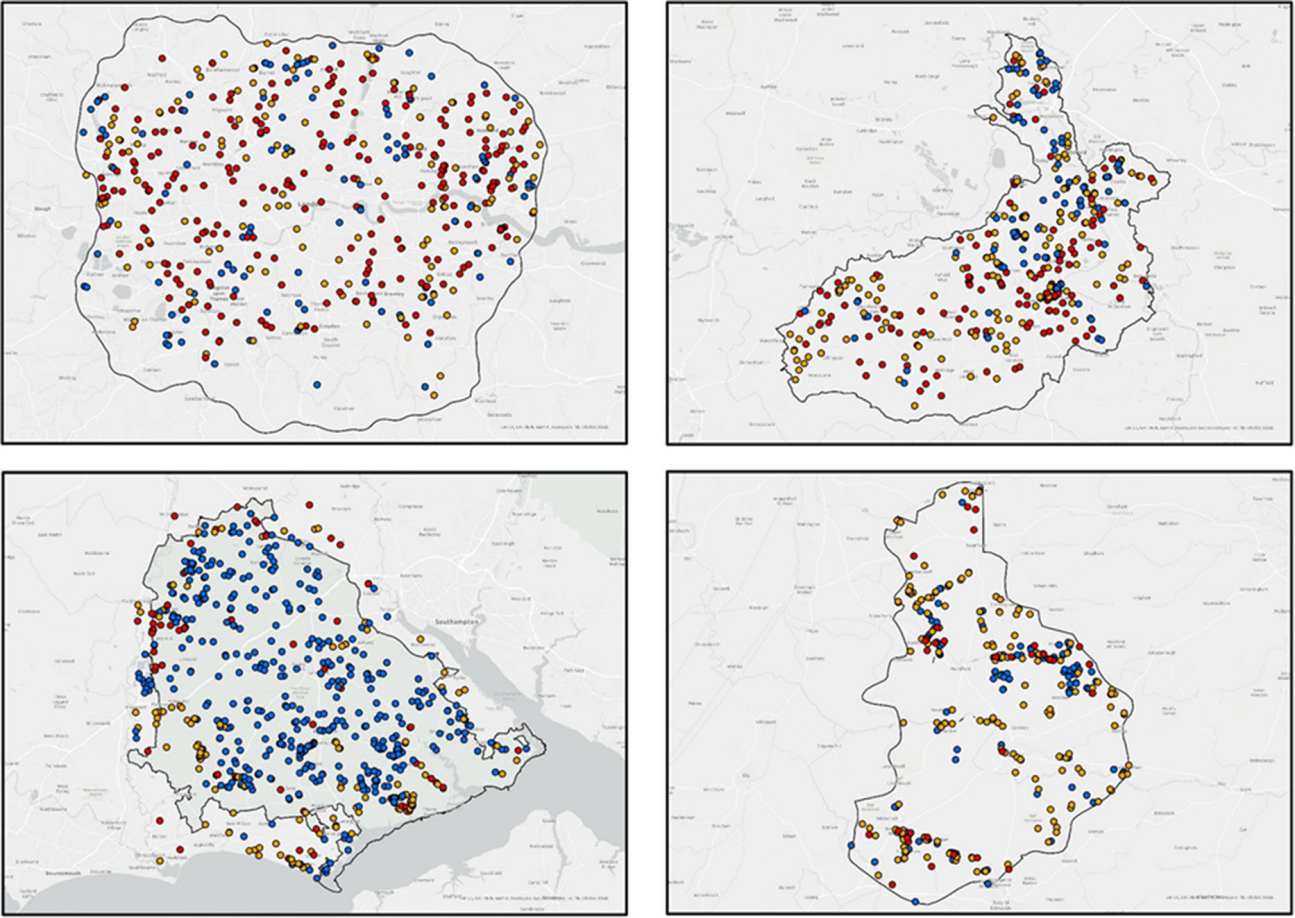
Results of whole landscape evaluation of nutrient pollution by citizen science in four contrasting landscapes in the UK. The results demonstrate contrasting water quality patterns in the four demonstration landscapes. In Greater London, there are a surprising number of clean standing waters, mainly ponds, in a landscape where running waters are near universally polluted (**a**). In the River Ock catchment (**b**), a typical lowland England mixed intensive farming and urban landscape, there are isolated clean water patches in ponds, some headwater streams and lakes. The largely clean water landscape in the New Forest National Park (**c**) reflects the predominant semi-natural, very low input, traditional farming landscape, whereas in The Breck (**d**), a mixed intensive farming/high nature value landscape, again, clean water is largely restricted to smaller waters, standing and flowing, with near universal pollution of the large rivers

‘Clean Water for Wildlife’ data have been collected at a number of scales including national and regional surveys. From 2012 onwards around 1500 volunteers have collected data throughout England and Wales, with a special focus on four key demonstration landscapes: Greater London (McGoff et al., 2017), the catchment of the River Ock, the New Forest National Park and The Brecks (Fig. 2a–d) (Ewald et al., 2018). This has involved data collection from approximately 5900 sites, including about 500 sites in each of the focal landscapes. All types of freshwaters (ponds, streams, lakes, rivers, ditches, etc.) are included, with the emphasis on smaller waters, with 45% of records from ponds, 32% from streams and ditches and the remainder from larger waters.

#### Lessons learned

Overall, the ‘Clean Water for Wildlife’ data, mostly collected by citizens, provide some of the first evidence of the overall pattern of water pollution in whole landscapes and the role of small waters in providing a reservoir of clean water. Comparing the rural and urban landscapes, results from the ‘Clean Water for Wildlife’ surveys show, perhaps surprisingly, that when account is taken of smaller waters, landscapes dominated by urban and suburban land use support similar proportions of clean water bodies, in terms of nutrient pollution, to those seen in typical intensively cultivated rural landscapes.

In terms of participation, the PACKTEST kits used in ‘Clean Water for Wildlife’ are highly effective and easy to use. However, training and quality control remains important, as in all surveys. Perhaps the most important lesson to be learnt is the recognition of the importance of survey administration and support for volunteers: it is easy to overlook the amount of effort involved in setting up a survey designed to obtain representative results (e.g. stratified random), permissions to visit sites, supporting data collection and reporting findings to volunteers. Working with volunteers also brings specific requirements and limitations in terms of health and safety. Hence, volunteer-based surveys can take as much time as professionally organised surveys: survey organisers and funders must be realistic about the pros and cons of such surveys, and be careful to avoid presenting citizen surveys as a low-cost alternative to professional surveys.

#### eDNA application

The use of environmental DNA to monitor freshwater biota has grown explosively over the last few years (e.g. Jan Pawlowski et al., 2018) and some uses are potentially suited to non-specialists. One of the original citizen applications of eDNA was the assessment of great crested newts which are a protected species in the European Union. Biggs et al. (2016) developed and tested the use of eDNA for detecting this species as a ‘citizen’ science tool, with eDNA expertise provided by the Spygen company in France. Non-specialists were sent to sites across Britain to determine whether citizens could collect eDNA data effectively, and both professional and citizen validation studies compared DNA with traditional great crested newt sampling methods (Biggs et al., 2016). In the UK, surveys of great crested newts using eDNA are run by FHT under the banner of the PondNet programme, a suite of surveys and data collection initiatives designed for use by volunteers although applicable to professional surveyors also.

This great crested newt programme proved remarkably successful and kick-started large programmes of testing for great crested newts in the UK, much of which is now undertaken professionally. This reflects the fact that great crested newt conservation is a well-funded process in Britain with many professional consultants involved, and eDNA being a financially beneficial survey technique for professionals. Nevertheless, for citizen programmes the costs of eDNA tests still remains a barrier.

Following the first national pilots in 2013, and the first national surveys in 2015, the great crested newt national monitoring programme has continued with a hybrid model of both professional and unpaid workers collecting eDNA samples and laboratory analyses undertaken in several different laboratories.

Overall, the collection of eDNA data by citizens appears to be currently limited and particularly for rivers where eDNA transport by flow complicates interpretation. However, as methods and genomic databases improve and costs reduce, eDNA data from citizen science initiatives could provide valuable data on species richness and distribution including the occurrence of invasive species (Huddart et al., 2016) which are relevant for small water bodies.

### Anglers’ Riverfly Monitoring Initiative (ARMI)

#### Background and methodology

ARMI, a nationally coordinated project, was officially launched in the UK in 2007 as a means for citizen scientists to assess river water quality based on the abundance of certain key invertebrate taxa (Brooks et al. 2019). The ARMI protocol is a simplified version of the routine biomonitoring methods used by the regulatory agencies in the UK. Volunteers use a standard sampling net (250-mm frame and 500-mm deep net bag with 1-mm mesh) to sample each site once a month throughout the year, by taking a 3-min kick sample from the range of habitats present at the site. The volunteer then spends 1-min selecting large stones from the riverbed and hand-wiping them in the mouth of the net to dislodge organisms which may not have been collected in the kick sample. The volunteer subsequently cleans the net contents of fine silt, empties the remaining contents into a sorting tray, and estimates the log_10_ scale abundance of 8 macroinvertebrate target taxa. These taxa [cased Trichoptera, caseless Trichoptera (caddisfly), Ephemeridae (mayfly), Ephemerellidae (blue-winged olive), Baetidae (olives), Heptageniidae (flat-bodied mayfly), Plecoptera (stonefly) and Gammaridae (shrimp)] were chosen because they are easy to identify at this taxonomic resolution, cover a range of sensitivities to pollution, have national applicability, are present year-round (with the exception of Ephemerellidae), and are familiar to most anglers. An ARMI score for the site is then generated by allocating a score of 1–4 according to the log_10_ abundance category of each target taxon and summing the scores of all the target groups. Local regulatory authorities give each sampling site a trigger level score based on their long-term data for the site. Higher ARMI scores indicate higher river quality, and the trigger level is set significantly below the expected ARMI score for the site. An ARMI score below the trigger level will therefore indicate that a serious pollution incident may have occurred. If the trigger level is breached, the ARMI volunteer confirms the breach by resampling the site and then informs the local agency officer who will investigate the cause and take appropriate action.

#### Training

Prior to participating in the ARMI scheme, the volunteers are trained in the ARMI protocol at a 1-day workshop. Training is conducted by an accredited tutor, and the local regulatory authority officer usually attends and assists with the training. Each participant is given a laminated fold-out chart that provides a simple identification guide to the 8 target invertebrate taxa, a description of the sampling protocol and scoring mechanism and instructions on what to do if the trigger level is breached. The chart also includes information on how to upload results to the national ARMI database and guidelines on health, safety and biosecurity. The training workshop is usually located at a venue close to the site the volunteers will monitor and consists of a classroom session in the morning and a practical session on the river in the afternoon. When a volunteer has successfully completed the training workshop, they are issued a certificate.

Volunteers are organised locally by a river coordinator, and river groups are usually associated with local hubs who coordinate river groups on a catchment basis. River coordinators upload their data onto the national database held at the Freshwater Biological Association, although some choose to hold their records locally.

#### Data capture and extent of results

Thousands of volunteers have been trained since 2007 and records from over 2,800 sites throughout the UK are currently stored on the national database. Headwaters are included by ARMI volunteers in some areas of the country but they are not specifically targeted. A total of 36,700 records are currently held on the national database. From January 2020 to July 2021, during a period of covid 19-related lockdown when relatively few samples were taken by the Environment Agency and volunteer activity was generally reduced, ARMI volunteers uploaded 3778 records to the national database, capturing information from 757 sites, on 349 rivers, across 105 catchments and highlighted 197 trigger level breaches. This underlines the value of ARMI as a ‘neighbourhood watch’ scheme in which volunteers were keeping an eye on the river when the statutory agency staff were largely absent.

#### Lessons learned

For quality assurance, it is essential that volunteers rigorously adhere to the sampling protocols. This is to ensure data quality, to reassure data users that the volunteer-generated data are reliable, and to minimise false alarms which may waste the time of investigative agency staff. The importance of carefully following the sampling protocols is emphasised during training of volunteers and tutors. The same training programme is used throughout the project to ensure all volunteers receive identical training. The national project manager maintains, develops and expands the ARMI network by setting up training workshops in areas that currently have poor ARMI coverage.

In order to maintain volunteer engagement, it is essential that they receive prompt and informative feedback from their ARMI coordinators and are kept in touch with national developments of ARMI through newsletters, and local and national meetings. Similarly, feedback from Environment Agency ecology officers detailing their responses to trigger level breaches reassures volunteers that their reports are resulting in positive action. Volunteer motivation is promoted by the knowledge that they are participating in a larger national organisation that is demonstrably helping to improve river water quality.

The development of the various ‘Riverfly Plus’ packages also helps to motivate volunteers by providing progressive learning outcomes, and enabling volunteers to understand more about river ecology and the impacts of stressors on the river environment.

A streamlined, easy to use and functional database and website ensures a more efficient use of Riverfly Partnership staff time and encourages volunteers to input and store their data on the national database. The national database allows volunteers to access their data in graphical form, and they can compare their records with others around the country. Annual and long-term data on national trends, information on numbers of active volunteers, numbers of actively monitored sites and numbers of trigger breaches and trigger-breach hotspots, which are useful for reporting statistics, are also reliant on wide use of the database by volunteers.

### RiuNet and Projecte Rius

#### Background and methodology

The RiuNet (www.riunet.net) and Projecte Rius (www.projecterius.cat) projects were initiated by the Ecology Department of the University of Barcelona. The main difference between these two projects is the commitment required of the citizen. RiuNet can be applied where and when one wishes to survey. In contrast, Projecte Rius has a network of volunteers that study the same river reach two times a year, in spring and autumn, to provide data from Mediterranean rivers in the high and low flow periods.

Both deploy similar methods for assessing river water quality, using macroinvertebrates as bioindicators, riparian forest quality and instream habitat heterogeneity with simplified protocols in the RiuNet handbook (https://www.ub.edu/fem/docs/Riunet/RiuNet_NOU_thebook_ENG.pdf). In terms of macroinvertebrates, users may recognise up to 40 different organisms, usually identified at family level, using pictures or the app's dichotomous key. Most organisms are insects: Diptera, Coleoptera, Odonata, Heteroptera, Trichoptera, Ephemeroptera and Plecoptera. However, they also consider some taxa from the Mollusca, Oligochaeta, Hirudinea and Crustacea. Each indicator has its value of sensitivity or tolerance to pollution or impacts based on the IBMWP index, the official biological index for Mediterranean rivers of the Iberian Peninsula (Alba-Tercedor & Sánchez-Ortega, 1988). The RiuNet app automatically calculates a simplified biological quality index (from 1 to 10),

The quality of the riparian forest was defined by Munné et al. (1998) and the heterogeneity of fluvial habitats (IHF) by Pardo et al. (2002). In RiuNet, both methods were combined in the so-called hydromorphological quality assessment. It consists of eight questions where users have to select different options comparing the study site with available pictures, and a list of features is provided for each question to enable selection of the closest match. Five questions are asked about the riparian forest characteristics: (a) is there native or non-native vegetation? (b) is the vegetation continuous or in isolated clutches? (c) is there is good connectivity with the nearby landscape? (d) are there are channel modifications? and (e) is litter present? Three questions are asked about aquatic habitat heterogeneity: (a) how many hard substrata are present (b) if there are areas with different depths and (c) velocities of the water, and how many types of organic substrata are present in the study site. Each answer has a score that is summed to give the hydromorphological quality: 40–36-very good; 35–29-good; 28–21-moderate; 20–11-bad and 10–0-very bad. Projecte Rius has similar questions to describe the study site’s riparian forest and aquatic heterogeneity, but no indices are calculated. Both projects have methods to validate results and to share the data on a map on their websites. In addition, Projecte Rius also produces an annual report of activities and results.

#### Training

Projecte Rius has a training programme for volunteers whilst RiuNet does not. Instead citizens use the project’s mobile app, an interactive educational tool that guides any citizen in diagnosing the hydrological and ecological status of a river.

#### Data capture and extent of results

**Fig. 3:**
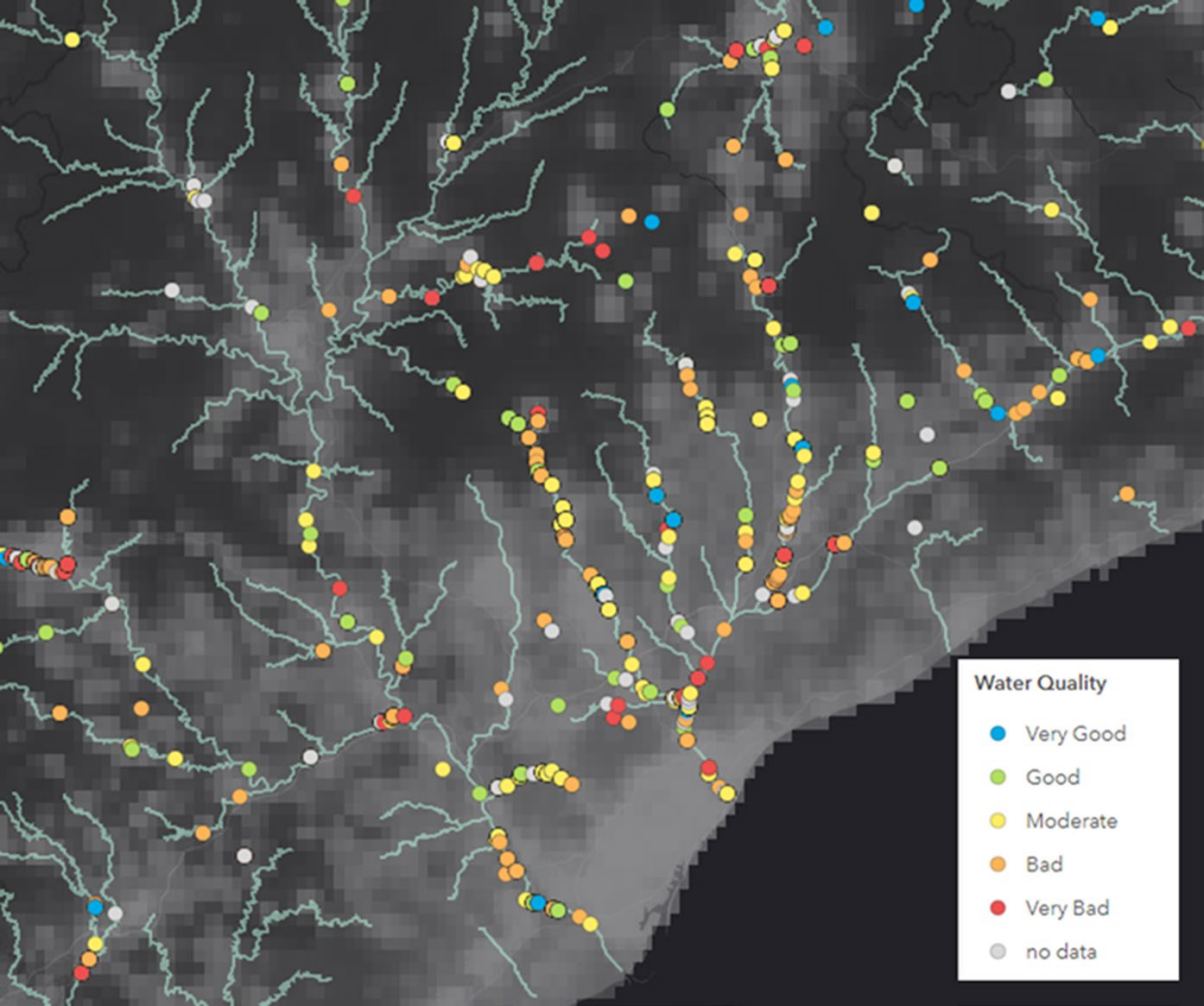
Map of the Barcelona region (NE Spain) with the studies of macroinvertebrate-based assessment of water quality undertaken by volunteers of Projecte Rius from 2005 to 2020. Blue lines represent the main river network (rivers or streams considered as water bodies by the Catalan Water Agency). The map shows that only a few studies are in small streams outside of the main river network. Base Map: population density (WorldPop, 2018). Grey: high population density; black: low population density. To see this map on a website: https://arcg.is/1q94zG

In these projects, educators, scientists, managers and water stakeholders work together to enable more efficient collection of data. Educators target environmental educational activities, often organised as festival and other events conducted by municipalities, schools, community centres, or NGOs in cities located near rivers and streams. Thus, most data are from highly populated areas in Spain, usually in valleys, plains, or near the coast, which means that most of the studies are not undertaken in small water bodies. For instance, Projecte Rius has a dataset of 3777 studies undertaken between 2005 and 2019 in Catalonia (NE Spain), however, to date only 122 studies have been in small water bodies (see example from the Barcelona metropolitan area, macroinvertebrate-based assessment of water quality Fig. 3). Data are uploaded on an online form on the project website or via the mobile app.

#### Lessons learned

As educational initiatives, these projects were, and are, very successful, especially in Catalonia (Spain) with thousands of people involved. The data collected add a new layer of information that can be used to better manage freshwater ecosystems. As noted, few sites on small water bodies are visited. In conclusion, there is a need to enhance the potential of Projecte Rius and RiuNet to be used to study small water bodies, and in consequence, provide new information on macroinvertebrate biodiversity, riparian forest quality, or hydrologic impacts in sparsely populated areas.

### WaterBlitz—e.g. Dublin WaterBlitz

#### Background and methodology

In 2015, Earthwatch Europe ran their first Freshwater Watch WaterBlitz on the Thames (UK), to engage citizens within the Thames basin collecting as many water quality data points as possible over a four-day period. In 2019, this was extended out to other European cities, including Dublin. Due to the restrictions caused by COVID-19, no WaterBlitz was run in 2020. In May 2021, the WaterBlitz was again run over four days in the Thames (UK) and in the Greater Dublin Area (Ireland). The Dublin WaterBlitz was led by the Dublin City University (DCU) Water Institute.

Nitrate and phosphate were measured in freshwater sites chosen by citizen scientists across the city of Dublin and surrounding areas over the 4-day period. The water was collected from local streams and water bodies using sampling devices created by the participants, such as a clean bucket attached to a rope, or a cut-away plastic bottle attached to a bamboo stick. These sampling devices were rinsed with river water before taking the test sample to avoid contamination. Participants were provided with sampling cups to ensure that all participants used the same volume of water. Testing of nutrients took place in situ using nitrate (NO_3_-N) and phosphate (PO_4_-P) Kyoritsu PackTest (Kyoritsu Chemical-Check Lab., Corp., Yokohama, Japan) water chemistry kits supplied by FWW (described above).

#### Training

**Fig. 4:**
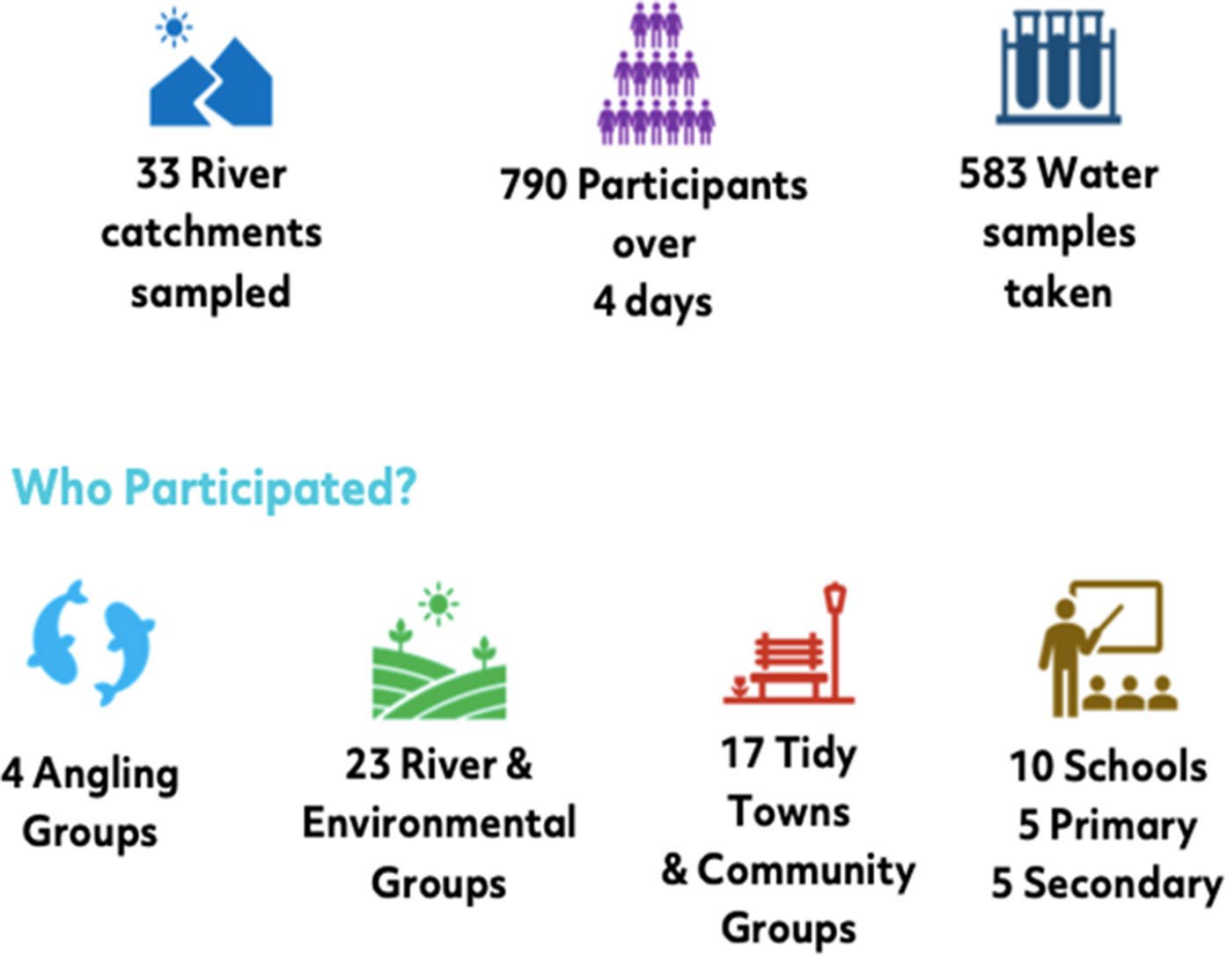
Illustration of the numbers involved in the Dublin and environs WaterBlitz (May 2021)—Citizen Science activities for Nutrient Monitoring, highlighting community groups that participated

As part of the Water Blitz in Ireland just under 800 volunteers were trained via the pre-existing FWW citizen science programme (https://freshwaterwatch.thewaterhub.org/). This training assured that all participants were using the same methodology. After conducting the tests, participants added their data to the FWW database, including observational notes or photographs of any of the following: presence of algae, surface foam, oily sheen, pollution discharges, litter and signs of aquatic life. Location coordinates were entered manually or by map geolocation from the smart device to the database on the FWW website or smartphone application. Many of the volunteers were involved in pre-existing environmental groups (Fig. 4), which facilitated both training and organisation of the Dublin WaterBlitz.

**Fig. 5:**
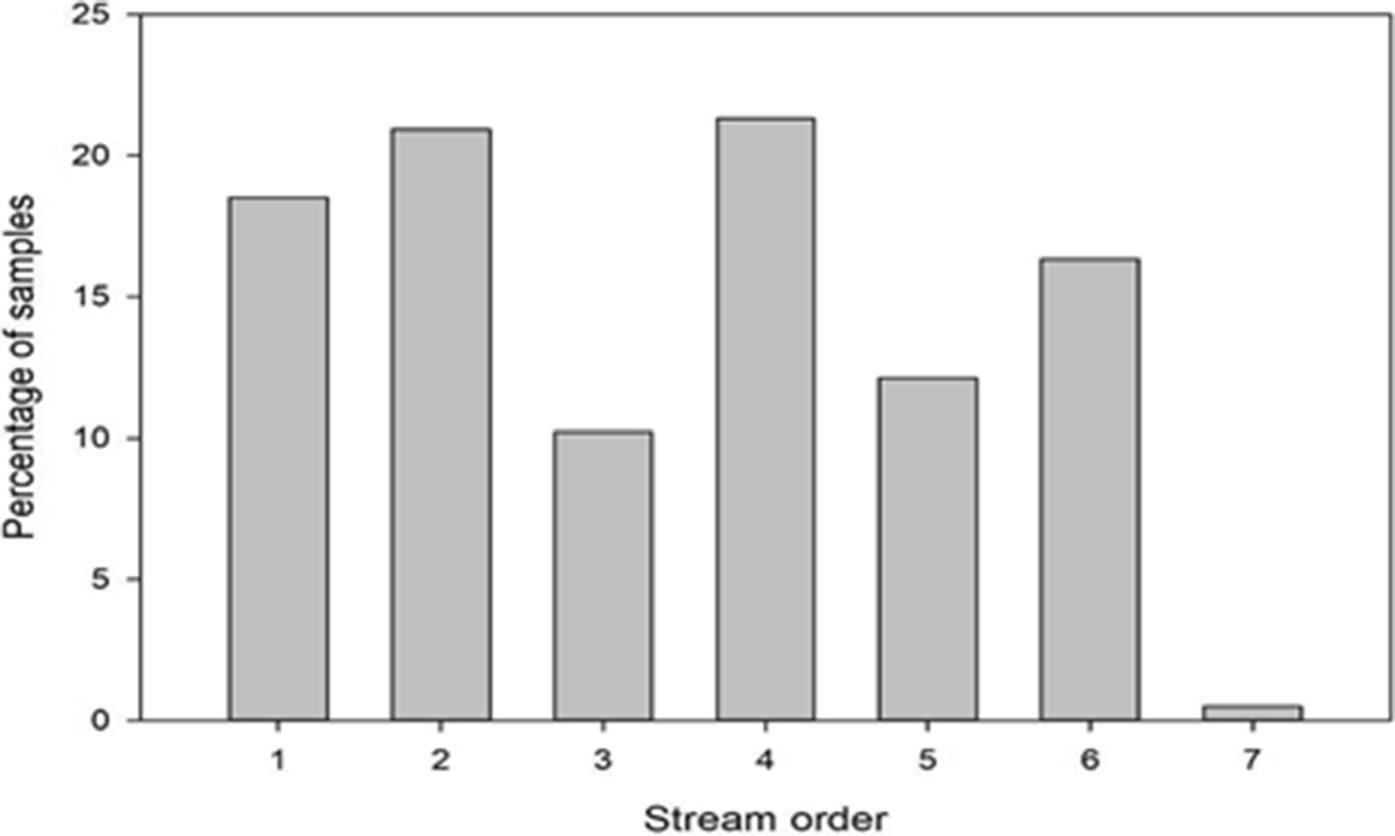
Percentage of samples taken from streams and rivers during the Dublin WaterBlitz of May 2021. Stream order is as per EPA (Ireland) categorisation

Over the course of the four-day WaterBlitz, nitrate and phosphate levels were measured in over 33 catchments by citizen scientists who sampled at 583 sites. The participants were encouraged to monitor their local stream/river and although a variety of stream orders were monitored, 33% of all sites sampled were in first or second order streams (Fig. 5). All land-use types, from agricultural areas and forestry to urban parks and residential areas, were represented in the sampled sites.

#### Lessons learned

The data from the Dublin WaterBlitz indicated the potential of citizen science to fill gaps in data on lower order streams that are not currently included in monitoring programmes. It also showed the desire of local communities to become involved in a robust, science-based monitoring programme. The engagement of pre-established local community groups in particular was a feature of the Dublin WaterBlitz in 2021 (Fig. 4). The engagement from these community groups ensured that sample kits sent to participants were used for the project and that data were fed back through the app. Many groups self-organised, distributing material and ensuring sufficient sampling coverage of their area, thus bringing down the costs of the WaterBlitz for the DCU team, and ensuring a spatial distribution of the samples that was of benefit to both the local community and the researchers. This collaboration between the researchers and local communities ensured success of the event.

## Moving forward—a framework for operationalising citizen science in small water bodies

Volunteers are becoming increasingly involved in environmental research (e.g. Silvertown, 2009) including river environments (e.g. Di Fiore and Fitch, 2016) with citizen science contributing to what has been described as a ‘research revolution’ (Roberts, 2016). The spatial and temporal coverage of data collected by citizens in the small stream network and other small water bodies has the potential to greatly exceed that of other means of monitoring. However, a framework that captures the essential elements of effective citizen science is needed to ensure both the sustainability of volunteer engagement and data quality. As noted in the examples provided, data quality depends upon using simple, clearly defined, validated methods to reduce operator variance to acceptable levels (Bird et al., 2014).

**Fig. 6:**
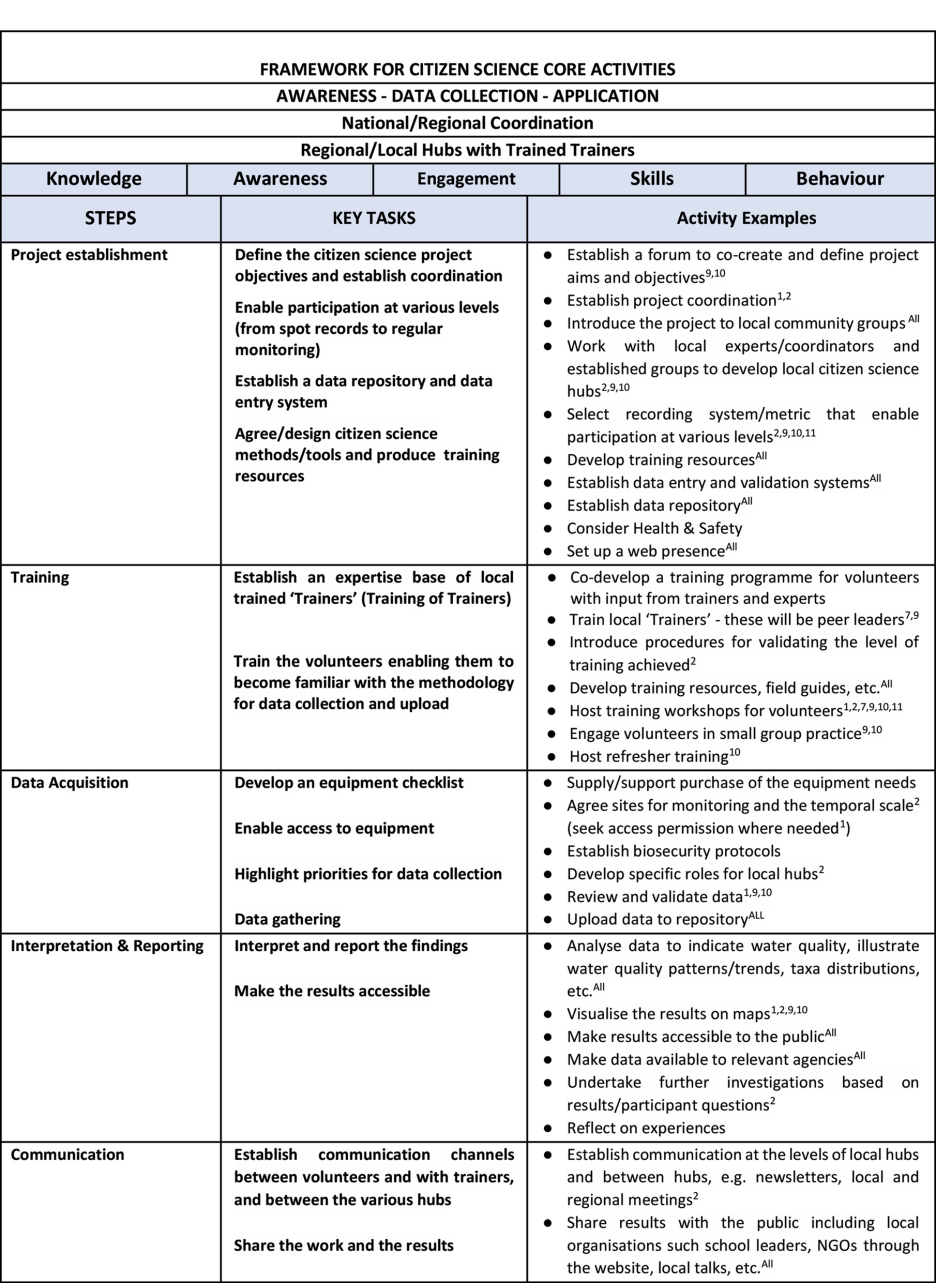
A framework for operationalisation of citizen science monitoring of small water bodies. Selected examples of projects from Table 1 with the listed elements are indicated using superscripted numbers

Based on the experience of the case studies described in this paper and published work (Jollymore et al., 2017; Gurnell et al., 2019), a framework for the establishment and implementation of citizen science for monitoring of small waters is proposed. Whilst this paper is focussed on small water bodies, the framework is relevant to citizen science projects more generally. Figure 6 outlines the “key attributes” of the Framework. A primary objective of a citizen science project is data collection, however, the potential to raise awareness about degradation of water quality and biodiversity loss, is important when creating the project. The experience of long-running projects, such as those covered in this paper, highlights the need for a defined organisation at national or regional level taking responsibility for overall coordination. Such a body would have oversight of the project's objectives and develop a strategy to enable a network of regional or local hubs, as well as address issues relating to site access, health and safety and data management.

Sustaining engagement and data collection is the most challenging task and can be helped by effective coordination and communication between coordinators and volunteers, and between volunteers (local hubs help in this respect). Other issues causing attrition amongst trainers as well as trained volunteers, such as the availability of resources (both financial and teams of experts to train trainers) need to be considered, as highlighted by Mormina & Pinder (2018). Projects funded under specific schemes usually have a limited life and all the training and interest can be lost when they end. Significant resources are required to recruit, train and support citizen scientists and quality control the data they collect. Whilst citizens can fill spatial and temporal gaps in information at a low cost, it is important that relevant agencies recognise the value of such data and, whenever possible, provide financial support to such data gathering activities.

Design of projects that facilitate varying levels of participation in the project and in data collection should be considered. At the project level, participation can be structured in three main categories, (1) *contributory* where participants collect data in projects designed by scientists; (2), *collaborative* where participants provide data, but may also help with project design, data analysis or dissemination of findings and (3) *co-created* where participants engage in the design and all stages of the project (Miller-Rushing et al., 2012; Thornhill et al., 2019). Most projects to date have been largely contributory in nature, but there is the potential for well-established projects to better integrate input from trained volunteers. Whilst access to gathered data and mapping of the data provides some feedback, enabling citizens to comment on methods used, sampling designs, improvements that could be made to a survey, and interpretation of results all help to embed citizens in the project and allow them to contribute to driving the science and its outcomes. This is what is meant by co-creation and few citizen science projects achieve this.

In terms of data collection, schemes that are adjusted to varying levels of expertise (e.g. CSSI and SSIS schemes in Ireland and the’Riverfly Plus’ in the ARMI project) help maximise participation and allow progression as interest, knowledge and skills develop. Capacity building of teams of volunteers for the long term should consider training of trainers (ToT), known as the cascade approach. This approach has been widely and successfully used in many sectors and typically involves external experts training a group of local trainees in specific technical skills and how-to train others in that subject. It enables relatively quick upskilling because it focuses on the practical and knowledge skills deficits of the trainees. The TRAIN framework of Mormina & Pinder (2018) provides a useful structure with which to plan for sustainability of a ToT programme. The trainers should have the opportunity to learn how to conduct a survey or identify indicators, and to learn about the ecology and the threats to the water bodies that they are surveying. This will help create greater awareness of environmental issues, one of the objectives of citizen science projects, and help foster engagement.

Educational material and tools are obviously essential, and these must be geared to the level of expertise of the participants. Examples range from identification sheets and manuals to phone applications. As noted by Graham et al. (2011) smartphone technology and mobile apps have the potential to revolutionise the collection of citizen science data and enable participants to have access to their own data and data from others. A wide range of other technologies are available to enhance engagement and data collection (Kristensen & Walley, 2018; Mazumdar et al., 2018). However, it is critical that participants are adequately trained in indicator identification before using app-based identification tools.

It is widely recognised that citizen science data are underutilised because of, often unfounded, concerns about data reliability (Bonney et al., 2014). It is thus essential that citizen science projects have a system of screening and validating data before they are made available to the public. Importantly, data collected by citizen science must be used and made available publicly, ideally through the project’s website using approaches that best visualise the results. Some results, such as in the ARMI, trigger resampling to confirm results and/or provoke action by responsible agencies.

Finally, effective communication channels between participants and coordinators, and between participants within and between hubs must be established early in the project. This is a critical, but is often an underestimated element of successful citizen science projects. The project also needs to be visible to the general public and this can be achieved through a web presence and regular blogs etc. shared through social media. As noted by Vohland et al. (2021) the project needs strong individuality including well defined visual identity (logo, etc.) and clearly defined goals to capture people’s attention. Communication of the project goals and results to local stakeholders and the general public helps spark interest and creates awareness, and promotes volunteer engagement.

In summary, there is an identified need to obtain better spatial and temporal coverage of water quality, biodiversity and the physical habitat condition of small water bodies. Citizen science can help address the information gaps, but the effort required should not be underestimated if such projects are to be sustained and to generate reliable and sustained data collection. A vision for sustainable citizen science should involve co-creation of projects and long-term monitoring under a defined supportive framework as illustrated in this paper.

## Data Availability

The data generated and analysed in this publication are available from the corresponding author upon reasonable request.
